# Utilizing the renal and vertebral contours as landmarks for right adrenal vein localization in primary aldosteronism: a retrospective analysis of 310 cases

**DOI:** 10.3389/fendo.2024.1505349

**Published:** 2025-01-08

**Authors:** Wei Sha, Yun Du, Shunkang Rong, Yuanqing Yao, Bo Xiong, Han Liu, Jun Qian

**Affiliations:** Department of Cardiology, The Second Affiliated Hospital of Chongqing Medical University, Chongqing, China

**Keywords:** primary aldosteronism, adrenal vein sampling, hypertension, catheterization, adrenal disease

## Abstract

**Background:**

Adrenal Vein Sampling (AVS) is the gold standard for categorizing primary aldosteronism (PA). However, catheterization of the right adrenal vein (RAV) can be technically challenging. This study aimed to investigate the validity of the right renal vertebral contour as fluoroscopic landmarks to help RAV orifice localization during AVS.

**Methods:**

Imaging data of 310 PA patients were retrospectively analyzed. Patients were divided into Normal, Overweight, and Obese group based on their body mass index (BMI). A novel Renal-Vertebral-Angle (R-V-A) model was employed to delineate the distribution of the RAV orifice. This model concerned a cruciate cross formed by the upper edge of the right renal and the right edge of vertebral contour under fluoroscopy. The area within a 2 cm×2 cm square in the left upper quadrant of this cross was defined as the R-V-A. The success rate of AVS was compared across different BMI groups, as well as the differences in the distribution of the RAV orifice within the R-V-A.

**Results:**

Successful RAV sampling was achieved in 270 cases, while the success rate of RAV sampling was found to be lower in the Obese group. The majority of the RAV orifices were located within the R-V-A region (249/270, 92.2%). There was no significant difference in the distribution of RAV orifices across the BMI groups (Normal vs. Overweight vs. Obese: 92.2% vs. 91.9% vs. 93.3%, *p*=0.968). In contrast to patients with successful RAV sampling, a significantly lower proportion of sampling site were found within the R-V-A in cases with mis-catheterized cases (92.2% vs. 55.6%, *p*<0.001).

**Conclusion:**

The R-V-A model could be utilized as an anatomical landmark for the RAV orifice localization on fluoroscopy, that might help to narrow down the exploration range for RAV catheterization, and might offer beneficial assistance in enhancing the success rate for AVS.

## 1 Introduction

Primary aldosteronism (PA) is one of the most common causes of secondary hypertension. Over 98% of PA patient present with unilateral aldosterone-producing adenoma or bilateral idiopathic hyper-aldosteronism (1). Accurate sub-typing of PA plays a pivotal role in selecting the most appropriate targeted treatment, either adrenalectomy when a unilateral adrenal form is ascertained or life-long medical treatment with mineralcorticoid recept antagonist in bilateral adrenal forms. Adrenal CT or MRI might be misleading and unreliable to distinguish unilateral from bilateral PA. Consequently, adrenal vein sampling (AVS) is regarded as the gold standard for localizing lesions of PA (2, 3).

However, AVS has a variable success rate and is a technically challenging procedure. Due to the small size and variable location of the orifice, sampling of the right adrenal vein (RAV) is often more challengeable. In most cases, the missing catheterization on the right side is the leading cause of the failure of bilateral AVS, that might require unconventional indices of lateralization (4–7). In our previous study, the right renal contour was utilized as a landmark for the RAV orifice localization under X-ray (8). However, the study was limited by a relatively small sample size and the exploration of the RAV orifice was confined to the vertical axis. Thus, we further reviewed the performance of AVS for PA at our center in recent 5 years, aiming to provide more precise reference information for the RAV orifice localization by exploration in a larger cohort.

## 2 Methods

### 2.1 Patients

This retrospective study was approved by Institutional review board of The Second Affiliated Hospital of Chongqing Medical University. The studies were conducted in accordance with the local legislation and institutional requirements. Given the retrospective design of this research, the need for informed consent was waived by our hospital institutional review board.

We included all patients diagnosed with PA who underwent AVS at our institution between Sep 2018 and September 2024. The diagnostic protocol for PA adhered to the established guidelines (9). Specifically, hypertensive patients with a positive aldosterone-to-renin ratio (ARR) [≥30 (ng/dL)/(ng/ml/h)] underwent the saline infusion test (2 L of isotonic saline administered with 4 hours). PA was diagnosed when plasma aldosterone concentration after saline infusion test ≥ 10 ng/dL.

Digital subtraction angiography (DSA) images and additional medical records of the enrolled patients were retrieved from medical record database server. All data were fully anonymized and de-identification, ensuring that researchers had no access to identifiable information. Personal numbers were scrambled to protect patients’ privacy.

### 2.2 AVS procedure

At least 2 weeks before AVS, the medication was switched to Diltiazem and/or Terazosin in all patients based on their blood pressure level.

AVS was conducted after one hour of resting in supine position between 8:00 am and 12:00 am via antecubital vein access, as previously reported (10). The procedure was performed without adrenocorticotropic hormone stimulation. Bilateral adrenal vein was cannulated sequentially using two types of catheters: a 5F MP for RAV sampling and a 5F TIG for left adrenal vein sampling. In cases where both sides of the antecubital vein access were unfeasible, the approach was shifted to the right femoral vein. If the RAV remained undetectable after 1-hour, further intervention would be discontinued in consideration of the risks associated with radiation exposure and potential complications.

The position of the catheter tip was confirmed by gently injecting a small amount of diluted contrast to reveal the angiography of the catheterized vein. The venograms at the anterior-lateral view was obtained at expiration period. If the cortisol concentration in the sampled vein was 2 times higher than that of the inferior vena cava (IVC), the AVS procedure is considered successful.

### 2.3 Angiography imaging analysis

Two investigators (Dr. Sha, and Dr. Du) independently reviewed and analyzed all imaging data using the Sante Dicom Viewer Free software (Version 5.3). If there is any dispute regarding the analysis results, the final decision would be made by Dr. Huang.

All patients were sub-grouped according to BMI, Height, and ARR, respectively. According to BMI, the patients were divided into the Normal group (BMI < 25.0kg/m²), Overweight group (25.0 ≤ BMI <30kg/m²), and Obese group (BMI ≥ 30kg/m²). Regarding height, they were divided into HEIGHT I group (Height 150 cm-169 cm), and HEIGHT II group (Height ≥170 cm). As for ARR, they were classified into ARR I group [ARR < 50 (ng/dL)/(ng/ml/h)], and ARR II group [ARR ≥ 50 (ng/dL)/(ng/ml/h)]. The cranio-caudal positioning of the RAV orifice was determined in relation to the vertebral bodies and discs under X-ray. Furthermore, expanding upon our previous research, we introduced a novel model termed as the Renal-Vertebral-Angle (R-V-A) to systematically explore the vertical-coronal axis locational characteristics of the RAV orifice.

**Figure 1:**
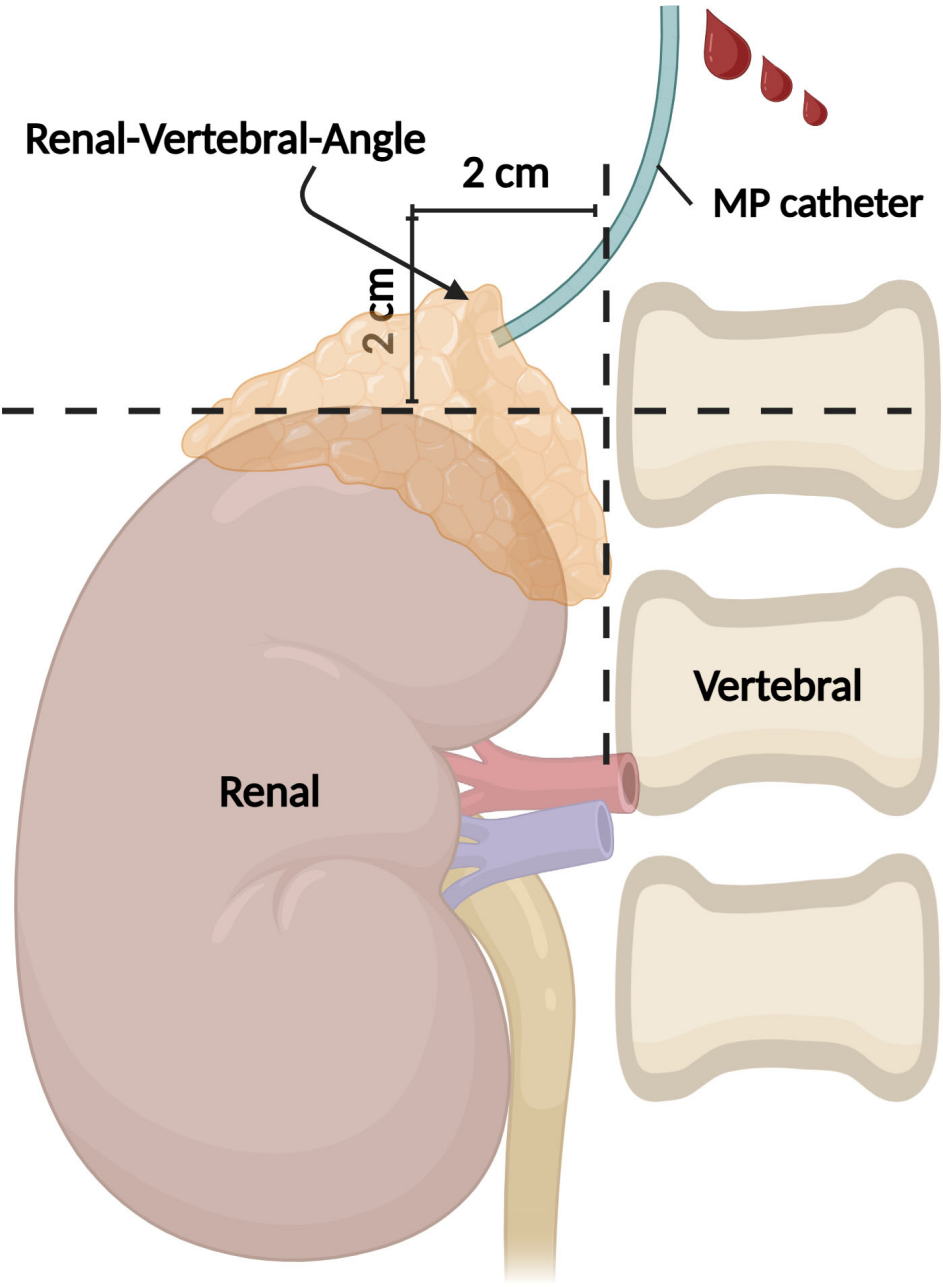
Illustration of the R-V-A. Under fluoroscopy, the upper edge of the right renal and the right edge of the vertebral contour formed an angle, and the 2 cm×2 cm area within the left upper quadrant of this angle is defined as the R-V-A. R-V-A, Renal-Vertebral-Angle. (Created in BioRender.com).

In detail, the methodology involved the depict of the RAV orifice by constructing a horizontal line from the upper edge of the right renal contour and a vertical line from the right edge of the vertebral body. The intersection of these lines formed a cruciate cross, while the area within the 2 cm×2 cm range of the left upper quadrant was defined as the R-V-A (**Figure 1**). The orifice of all sampled vessels, whether RAV or otherwise, were delineated inside or outside the R-V-A.

### 2.4 Statistical analysis

Normally or approximately normally distributed measurement data are presented as mean ± standard deviation. Enumeration data are expressed as percentages (%). To compare the success rate of RAV sampling and the distribution of RAV orifice within the R-V-A across different BMI, HEIGHT, and ARR groups, we employed the Chi-Square test or Fisher’s exact test, as appropriate. Meanwhile, the distribution of RAV orifice within the R-V-A across successful RAV sampling cases and mis-catheterization cases, was analyzed using the same statistical tests to account for any variations. Differences were considered as statistically significant if the *p* value was <0.05.

## 3 Results

### 3.1 Patients characteristics

Data from 310 PA patients who received AVS were analyzed. Clinical characteristics and laboratory parameters are summarized in **Table 1**, involving 143 males and 167 females with a mean age of 43.3 ± 12.3 years and mean ARR of 41.9 ± 26.7 {(ng/dL)/[μg/(L·h)]}. Over half of the patients exhibited adrenal thickening or nodules on CT scan (n=143, 54.5%). AVS was predominantly performed via the right antecubital vein (n=293, 94.5%), while 15 patients underwent the procedure via the left antecubital vein, and 2 patients via the right formal vein.

**Table 1:** Patients Characteristics.

Number of patients	310
Age	43.3 ± 12.3
BMI (Kg/m²)	25.9 ± 3.2
ARR{(ng/dL)/[μg/(L·h)]}	41.9 ± 26.7
Serum Potassium (mmol/L)	3.6 ± 0.9
Adrenal abnormal (thickening/nodules) on CT scan	143
24h MSBP (mmHg)	149 ± 17
24h MDBP (mmHg)	93 ± 13

BMI, Body Mass Index; ARR, Aldosterone-to-Renin Ratio; MSBP, Mean Systolic Blood Pressure; MDBP, Mean Diastolic Blood Pressure.

### 3.2 Bilateral catheterization success and failure analysis

A total of 266 cases of bilateral AVS were successfully performed (85.8%). As showed in **Table 2**, there was an association between BMI and the success rate of AVS. Individuals with higher BMIs showed a lower success rate in both bilateral sampling [Normal group: 114/133 (85.7%) vs. Overweight group 123/140 (87.9%) vs. Obesity group 29/37 (78.4%), χ2 = 91.026, p<0.001] and RAV sampling [Normal: 116/133 (87.2%) vs. Overweight: 124/140 (88.6%) vs. Obesity: 30/37 (81.1%), χ2 = 90.533, p<0.001]. However, there was no statistically significant difference observed in the success rate of RAV sampling across different HEIGHT (HEIGHT I vs. HEIGHT II: 169/191, 88.5% vs. 101/119, 84.9%, *p*=0.36) and ARR groups (ARR I vs. ARR II: 219/253, 86.6% vs. 51/57, 89.5%, *p*=0.55).

**Table 2:** Comparison of success rate of bilateral sampling and RAV sampling between different BMI groups.

Group	Normal	Overweight	Obese	Total	χ2	*p*
**Bilateral Sampling Success**	114/133 (85.7%) ^*^	123/140 (87.9%) ^*^	29/37 (78.4%) ^#^	266/310 (85.8%)	91.026	<0.001
**RAV Sampling Success**	116/133 (87.2%) ^*^	124/140 (88.6%) ^*^	30/37 (81.1%) ^#^	270/310 (87.1%)	90.533	<0.001

*indicates no significant difference between the groups; # indicates a statistically significant difference between the groups with *p*<0.001. RAV, Right Adrenal Vein; BMI, Body Mass Index. Bilateral Sampling Success means: "Bilateral adrenal vein sampling was successfully performed"; RAV Sampling means: "only right adrenal vein sampling was successfully performed".

A total of 44 cases experienced unsuccessful AVS procedures. Among these, 35 cases failed on the right side only, 4 cases on the left only, and 5 cases exhibited bilateral failure.

Among the 40 (35 + 5) cases of right-side failure, an analysis of the imaging data revealed that the RAV orifice was inaccessible in 10 cases. Misidentification of the RAV occurred in 18 cases, where the AHV or other unidentified veins were erroneously considered as the RAV. In another 10 cases, the venography indicated successful catheterization, which displaying a typical RAV pattern; yet the cortisol concentration in the samples did not exceed twice that of the IVC, which was potentially confounded by the dilutional effects of the IVC or AHV. Moreover, in 2 patients, the procedure had to be terminated early due to the development of a right adrenal hematoma, which was managed conservatively with analgesic medication.

Regarding the 9 (4 + 5) cases of left-side sampling failure, variant anatomy of the left adrenal vein was implicated 2 cases. In the remaining 7 cases, catheterization was potentially successful, yet the cortisol concentration in the samples did not exceed twice that of the IVC, possibly due to dilution by blood from the subdiaphragmatic vein.

Adrenalectomy or adrenal arterial embolization was recommended for all patients with lateralized aldosterone secretion. Patients with bilateral adrenal aldosterone secretion or those who did not achieve successful AVS were prescribed spironolactone or eplerenone.

### 3.3 RAV orifice location analysis

**Figure 2:**
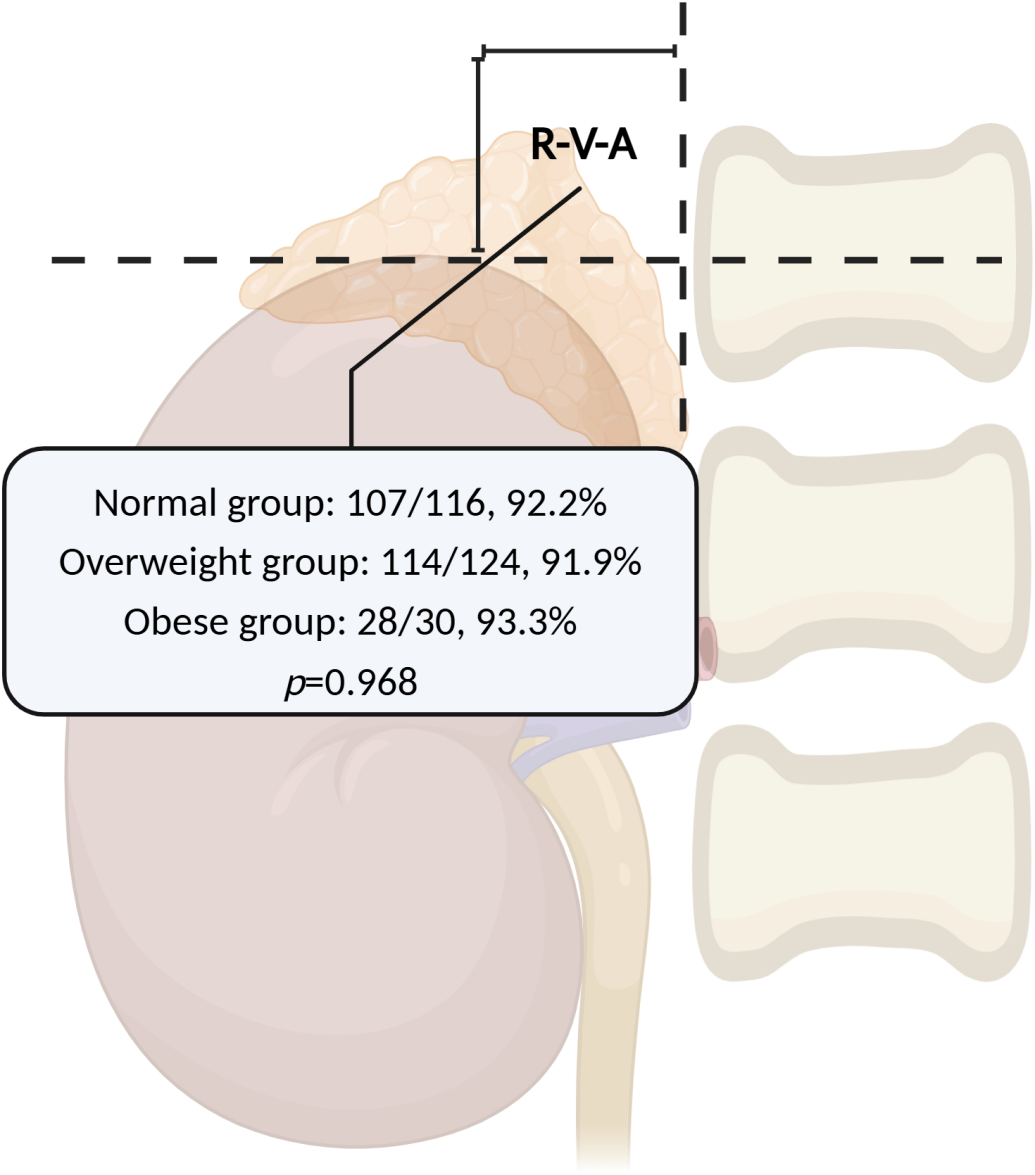
The RAVs orifice distribution within the R-V-A across different BMI groups. The proportion of RAVs orifice distributed within the R-V-A had no significant variance across different BMI groups in successful RAV sampling cases. RAV, Right Adrenal Vein; R-V-A, Renal-Vertebral-Angle; BMI, Body Mass Index. (Created in BioRender.com).

The right kidney contour could be visualized in 233/310 patients (75.2%) under X-ray scanning, which could be shown in the remaining patients by injecting a small amount of contrast medium (about 10-15ml). In all successful RAV sampling cases, the majority of RAV orifices were situated within the R-V-A region (249/270, 92.2%). Among the remaining 21 cases, 15 were distributed upon the R-V-A, 4 cases were on the left side, and 2 cases were below. The proportion of RAVs orifice distributed within the R-V-A had no significant variance across different BMI groups in successful RAV sampling cases (Normal: 107/116, 92.2% vs. Overweight: 114/124, 91.9% vs. Obese: 28/30, 93.3%, χ^2^ = 0.066, *p*=0.968) (**Figure 2**), as well as among different HEIGHT (HEIGHT I vs. HEIGHT II: 155/169, 91.7% vs. 94/101, 93.1%, *p*=0.69), and ARR groups (ARR I vs. ARR II: 202/217, 93.1% vs. 47/53, 88.7%, *p*=0.43).

Among the 40 failure RAV sampling cases, 18 incorrect sampling procedures were conducted, among which 8 sampling points were located outside the R-V-A, and 10 were within the R-V-A. Thus, the positive predictive value of localizing RAV within the R-V-A region is 96.1% (249/259). Retrospective analysis of the DSA revealed that 7 of these were accessory hepatic vein (AHV), while 1 was another undetermined vein. The proportion of sampling vessel orifice located within the R-V-A was significantly lower in mis-catheterization cases while compared to success RAV sampling cases (10/18, 55.6% vs. 249/270, 92.2%, χ2 = 25.054, *p*<0.001).

## 4 Discussion

AVS is recognized as the gold standard for lateralization diagnosis in PA in differentiating between unilateral and bilateral sources of autonomous aldosterone secretion. However, sampling from the RAV presents challenges, because its smaller size and variable orifice result in significant variations in AVS success rates across different centers (11, 12).

Our study revealed that the success rate of RAV sampling tends to be lower in individuals with a higher BMI. Several factors may underlie this observation: 1) patients with increased BMIs often present with a larger diameter of the IVC (13, 14), which might diminish catheter stability and supportiveness, particularly when employing the MP catheter for probing the RAV orifice, thus amplifying the procedural difficulty; 2) DSA imaging clarity might become less distinct in individuals with higher BMIs, potentially hindering the operator’s visual acuity.

Great efforts have been made to enhance the success rate of RAV sampling. Pre-operative enhanced CT scans might contribute to demonstrate localization of RAV orifice; however, the vessel is sometimes blurred in CT imaging, and not all the vessel can be detected (15, 16). The RAV sampling success rate could be arisen to 100% using C-arm CT (17), however, the C-arm CT is not available in all medical centers and might increase the medical cost and radiation exposure to patients.

Our prior research suggested that the right renal contour might be utilized as landmark for localizing the RAV orifice in a vertical orientation along the IVC (8). In order to comprehensively assess the location characteristics of the RAV orifice under fluoroscopy, we introduced the R-V-A model, which facilitated the exploration of the RAV orifice’s approximate distribution both in vertical and coronal orientation. Our results revealed a significant concentration of RAV orifices located within the R-V-A region in successful cases (249/270, 92.2%), which was partly aligning with the anatomical relationship of the kidney and the expected location of the adrenal gland. These findings suggest that targeting the RAV orifice within the R-V-A at the initiation of AVS procedure might be reasonable. Since almost all PA patients have routinely undergone adrenal CT examination before AVS, which provides approximate location information for the RAV orifices. Intraoperative combination of R-V-A model might provide a more efficient and accurate strategy for RAV exploration, effectively reducing the scope of exploration along the IVC. Notably, there was no significant difference observed in the distribution range of the RAV orifice among different BMI groups. This implies that utilizing the R-V-A model could be particularly beneficial for identifying RAV in obese patients, in which the success rate of RAV sampling is relative lower in the present study.

In contrast to patients with successful RAV sampling, a significantly lower proportion of sampling site were found within the R-V-A in cases with mis-catheterized cases (92.2% vs. 55.6%). In other words, this suggests that the likelihood of unsuccessful RAV sampling escalates when the catheterization site is positioned beyond the R-V-A. In such instances, careful assessment is warranted to ascertain the accuracy of the catheterization.

Meanwhile, it is crucial to emphasize that even in instances of mis-catheterized cases, over half of the sampling sites (10/18) were also located within the R-V-A. This finding underscores the necessity for meticulous differentiation from the RAV. In fact, encountering other veins during RAV probing is not an uncommon occurrence. Upon retrospective analysis of DSA images, we found the majority of these vessels were AHVs. The AHVs are particularly prone to misidentification with the RAV, potentially leading to sampling failure, especially when their orifices are in close approximation to the RAV (18). Drawing from our experience and previous studies, the AHVs typically exhibit parenchymal liver staining upon contrast medium administration, manifesting a nebulous appearance, in contrast to the sparser angiographic profile characteristic of the RAV (3, 19).

In conclusion, the results of the present study suggest that the majority of RAV orifices were localized within the R-V-A under X-ray. Although a few veins orifices with incorrect sampling were located within the R-V-A region, however, R-V-A remains a significant indicator for RAV detection with a high positive predictive value (96.1%) in this study. Given the above findings, using the right renal and vertebral contour as an anatomical landmark could help to approximately predict the RAV with different BMI, and help to narrowing down the probing range during AVS procedure.

## 5 Limitation

This study has several limitations. Firstly, the research is confined to a single-center and retrospective design, which may introduce biases. A prospective validation cohort study is needed in future time. Additionally, the sample sizes across the BMI categories are uneven, with a notably smaller cohort in the obese group, potentially skewing the statistical outcomes. What’s more, given that cannulation of the left adrenal vein typically poses minimal challenges in clinical practice, the present study’s emphasis was only placed on delineating the localization characteristics of the RAV orifice. Finally, the present study lacks multivariate analysis to identify additional predictors of RAV distribution in the R-V-A region. However, we intent to conduct further investigations into this matter in the future by accumulating more AVS cases.

## Data Availability

The original contributions presented in the study are included in the article/supplementary material. Further inquiries can be directed to the corresponding author.

## References

[1] Reincke M Bancos I Mulatero P Scholl UI Stowasser M Williams TA. Diagnosis and treatment of primary aldosteronism. Lancet Diabetes Endocrinol. (2021) 9:876–92. doi: 10.1016/S2213-8587(21)00210-2 34798068

[2] Vaidya A Hundemer GL Nanba K Parksook WW Brown JM. Primary aldosteronism: state-of-the-art review. Am J Hypertens. (2022) 35:967–88. doi: 10.1093/ajh/hpac079 PMC9729786 35767459

[3] Quencer KB. Adrenal vein sampling: technique and protocol, a systematic review. CVIR Endovasc. (2021) 4:38. doi: 10.1186/s42155-021-00220-y 33939038 PMC8093361

[4] Rossi GP Rossitto G Amar L Azizi M Riester A Reincke M. Clinical outcomes of 1625 patients with primary aldosteronism subtyped with adrenal vein sampling. Hypertension. (2019) 74:800–8. doi: 10.1161/HYPERTENSIONAHA.119.13463 31476901

[5] Zhong S Zhang T He M Yu H Liu Z Li Z. Recent advances in the clinical application of adrenal vein sampling. Front Endocrinol (Lausanne). (2022) 13:797021. doi: 10.3389/fendo.2022.797021 35222268 PMC8863662

[6] Parasiliti-Caprino M Bioletto F Ceccato F Lopez C Bollati M Voltan G. The diagnostic accuracy of adjusted unconventional indices for adrenal vein sampling in the diagnosis of primary aldosteronism subtypes. J Hypertens. (2021) 39:1025–33. doi: 10.1097/HJH.0000000000002700 33186324

[7] Parasiliti-Caprino M Bioletto F Ceccato F Lopez C Bollati M Di Carlo MC. The accuracy of simple and adjusted aldosterone indices for assessing selectivity and lateralization of adrenal vein sampling in the diagnosis of primary aldosteronism subtypes. Front Endocrinol (Lausanne). (2022) 13:801529. doi: 10.3389/fendo.2022.801529 35250861 PMC8888437

[8] Qian J Du Y Yang G Yao Y Xiong B Rong S. Use the right kidney contour as a landmark in adrenal vein sampling. PloS One. (2022) 17:e0263945. doi: 10.1371/journal.pone.0263945 36173999 PMC9521845

[9] Funder JW Carey RM Mantero F Murad MH Reincke M Shibata H. The management of primary aldosteronism: case detection, diagnosis, and treatment: an endocrine society clinical practice guideline. J Clin Endocrinol Metab. (2016) 101:1889–916. doi: 10.1210/jc.2015-4061 26934393

[10] Dong H Huang J Zhang Y Dong Y Liu M Yan Z. Adrenal venous sampling via an antecubital approach in primary aldosteronism: A multicenter study. J Clin Endocrinol Metab. (2023) 109:e274–9. doi: 10.1210/clinem/dgad433 37466201

[11] Maruyama K Sofue K Okada T Koide Y Ueshima E Iguchi G. Advantages of intraprocedural unenhanced CT during adrenal venous sampling to confirm accurate catheterization of the right adrenal vein. Cardiovasc Intervent Radiol. (2019) 42:542–51. doi: 10.1007/s00270-018-2135-5 30519725

[12] Omura K Ota H Takahashi Y Matsuura T Seiji K Arai Y. Anatomical variations of the right adrenal vein: concordance between multidetector computed tomography and catheter venography. Hypertension. (2017) 69:428–34. doi: 10.1161/HYPERTENSIONAHA.116.08375 28137990

[13] Taniguchi T Ohtani T Nakatani S Hayashi K Yamaguchi O Komuro I. Impact of body size on inferior vena cava parameters for estimating right atrial pressure: A need for standardization? J Am Soc Echocardiogr. (2015) 28:1420–7. doi: 10.1016/j.echo.2015.07.008 26272698

[14] Patil S Jadhav S Shetty N Kharge J Puttegowda B Ramalingam R. Assessment of inferior vena cava diameter by echocardiography in normal Indian population: A prospective observational study. Indian Heart J. (2016) 68 Suppl 3:S26–30. doi: 10.1016/j.ihj.2016.06.009 PMC5198879 28038721

[15] Matsuura T Takase K Ota H Yamada T Sato A Satoh F. Radiologic anatomy of the right adrenal vein: preliminary experience with MDCT. AJR Am J Roentgenol. (2008) 191:402–8. doi: 10.2214/AJR.07.3338 18647909

[16] Lee BC Chang CC Liu KL Chang YC Wu VC Huang KH. Evaluation of right adrenal vein anatomy by Dyna computed tomography in patients with primary aldosteronism. Sci Rep. (2016) 6:28305. doi: 10.1038/srep28305 27334209 PMC4917856

[17] Georgiades CS Hong K Geschwind JF Liddell R Syed L Kharlip J. Adjunctive use of C-arm CT may eliminate technical failure in adrenal vein sampling. J Vasc Interv Radiol. (2007) 18:1102–5. doi: 10.1016/j.jvir.2007.06.018 17804771

[18] Miotto D De Toni R Pitter G Seccia TM Motta R Vincenzi M. Impact of accessory hepatic veins on adrenal vein sampling for identification of surgically curable primary aldosteronism. Hypertension. (2009) 54:885–9. doi: 10.1161/HYPERTENSIONAHA.109.134759 19687347

[19] Kobayashi K Alkukhun L Rey E Salaskar A Acharya R. Adrenal vein sampling: tips and tricks. Radiographics. (2024) 44:e230115. doi: 10.1148/rg.230115 38662586

